# PLA 3D Printing as a Straightforward and Versatile Fabrication Method for PDMS Molds

**DOI:** 10.3390/polym15061498

**Published:** 2023-03-17

**Authors:** Guus van der Borg, Harry Warner, Melina Ioannidis, Geert van den Bogaart, Wouter H. Roos

**Affiliations:** 1 Molecular Biophysics, Zernike Institute for Advanced Materials, Rijksuniversiteit Groningen, 9747 AG Groningen, The Netherlands; 2 Department of Molecular Immunology, Groningen Biomolecular Sciences and Biotechnology Institute, Rijksuniversiteit Groningen, 9747 AG Groningen, The Netherlands

**Keywords:** polylactic acid, filament 3D printing, PLA, PDMS, mold, PLA smoothing, microscope chamber

## Abstract

3D printing is gaining traction in research and development as a way to quickly, cheaply, and easily manufacture polydimethylsiloxane (PDMS) molds. The most commonly used method is resin printing, which is relatively expensive and requires specialized printers. This study shows that polylactic acid (PLA) filament printing is a cheaper, more readily available alternative to resin printing, that does not inhibit the curing of PDMS. As a proof of concept, a PLA mold for PDMS-based wells was designed, and 3D printed. We introduce an effective method to smooth the printed PLA mold, based on chloroform vapor treatment. After this chemical post-processing step, the smoothened mold was used to cast a ring of PDMS prepolymer. The PDMS ring was attached to a glass coverslip after oxygen plasma treatment. The PDMS–glass well showed no leakage and was well suited to its intended use. When used for cell culturing, monocyte-derived dendritic cells (moDCs) showed no morphological anomalies, as tested by confocal microscopy, nor did they show an increase in cytokines, as tested using ELISA. This underlines the versatility and strength of PLA filament printing and exemplifies how it can be valuable to a researcher’s toolset.

## 1. Introduction

Polydimethylsiloxane (PDMS) is a commonly used material in biophysical and biomedical research, as it is optically transparent, non-fluorescent, non-toxic, and relatively impermeable to liquids [1,2]. In soft lithography approaches, it can be used as stamps or molds [3], for instance for the specific attachment of cells or cytoskeletal filaments [4,5]. Its most important feature is its prepolymer’s ability to conform to the contours of a mold with high fidelity [2,6]. These traits make PDMS an attractive choice of material when fabricating structures for use in studying biological systems. Therefore, it is widely used in the field of microfluidics, to construct flow cells, which sometimes include intricate surface features. Examples include, flow cells for electrochemical sensor fabrication [7], for culturing animal cells and cell adhesion studies [8,9], for studies of motor protein interactions with the cytoskeleton [10], and for flow lithography approaches to synthesizing hydrogel microparticles [11].

Before a PDMS structure can be made however, a mold is required. A mold is a negative of the desired structure and can be made of soft or hard material. Typically it is reusable, so with one mold, one can repeatedly make the same structure. Photolithography is classically the method used to fabricate molds for PDMS structures [3,5]. However, this process requires cleanroom access, microfabrication tools, extensive training, and expensive reagents. As an alternative, 3D (three-dimensional) printing can be used. Using this approach, also referred to as additive manufacturing technology, a 3D digital model is used to create a physical object, via layer-by-layer deposition of material at specific locations. In this manner, a real 3D physical object is built by successive addition of quasi-2D layers of material. In the scientific community, 3D printing is gaining traction as a way to relatively quickly, cheaply, and easily manufacture molds. As a technology, 3D printing is relatively accessible, with printers being commercially available at reasonable costs and little training required to use and design structures for printing. A variety of different materials can be used with 3D printing. For the fabrication of PDMS molds, resin or silicone are generally used [12,13,14,15]. However, resin and silicone are relatively expensive in relation to other 3D printable materials, and they require specialized printers. Another common problem of using resin printers for creating molds for use with PDMS, is that the resins tend to inhibit the curing of the polymer [14]. One of the cheaper alternative materials that is commonly used is polylactic acid (PLA). It is a polymer used for 3D printing in most commercially available, general-use filament 3D printers. PLA is a material that is cheap, biocompatible, and biodegradable [16,17]. It is used in the context of tissue engineering [18], medical implant applications [19], and drug delivery approaches [20]. PLA is easy to use for making a 3D print, and, in fact it is recognized by the USA-based Food and Drug Administration (FDA) as, generally recognized as safe (GRAS) [21]. In addition, PLA has the advantage that it does not inhibit the curing of PDMS. Despite this, and despite widespread use in other fields, PLA is not yet commonly used for molds. One problem that PLA printing encounters, is the far greater defined printing layers, which form rough edges on the mold, making them less suited for the purpose of creating PDMS molds. Yet, for less detailed prints, PLA can still be used. As PLA filament printing for the creation of molds is still relatively underrepresented in the scientific literature, this study aims to provide evidence that PLA filament printing is a suitable method of rapid prototyping in the fields of (micro)biological and biochemical studies. In order to make the molds suitable for use in the context of PDMS curing and flow-cell applications, we needed to introduce a post-manufacturing step. In particular, we present an improved approach, where we smooth the rough edges of the mold, making it suitable for polymer-casting. As a proof of concept, we designed and printed a mold for casting a PDMS-based well, to study biological samples by light microscopy.

## 2. Materials and Methods

### 2.1. Mold Design

Mold designs were made in Autodesk TinkerCAD (Autodesk, San Francisco, CA, USA). The design was then exported as an STL file, a 3D model file suitable for conversion to a 3D printable path, which was then sliced into a 3D printer path file, readable by the 3D printer, through the use of ReplicatorG (open source software). The STL files are added as Supplementary Information.

### 2.2. Printer and Settings

Molds were printed using a commercial 3D filament printer from Wanhao (Jinhua, China). In particular, the Duplicator 4 model of Wanhao was used. PLA filaments (3DNinja, Haarlem, The Netherlands) with 1.75 mm diameter were printed at 190 °C, on a Kapton tape covered metal plate heated to 60 °C. The layer height was set to 0.1 mm, and no rafts or supports were used. The print speed was 10 mm/s. 3D printing is also referred to in the literature as additive manufacturing or rapid prototyping. The term 3D printing is, however, nowadays typically used, and also fits with the terminology described by the ISO/ASTM 52900 standard terminology for additive manufacturing [22].

### 2.3. Mold Smoothing

Chloroform (99.0–99.4% purity, Merck, Rahway, NJ, USA) vapor was prepared by heating chloroform in a covered beaker in a fume hood. Printed molds were suspended in chloroform vapor using PTFE tubing four times, or until smooth, for 30 s, with a 1 min waiting time in between. After the smoothing, the molds were left suspended in the fume hood for a minimum of 1 h, after which they were placed in a vacuum desiccator overnight.

### 2.4. Mold and PDMS Preparation

The mold parts were assembled and kept in place with masking tape. PDMS was prepared with a base-to-catalyst mixture (9.1% *w*/*w*) of silicone elastomer (Sylgard 184; Dow Corning, Midland, MI, USA), poured into the mold, desiccated, and cured at 60 °C for at least 4 h. After curing, the mold was disassembled, and excess PDMS was removed using a scalpel, so the ring measured 3 mm in height.

### 2.5. Liquid Well Preparation

Glass coverslips were cleaned by sonication in acetone (>99.5% pure, Boom) for 30 min, followed by sonication in ethanol (>96% pure, Boom) for 30 min, and finally by 10 min of sonication in 1 M KOH (≥85% pure KOH pellets, Sigma-Aldrich, St. Louis, MO, USA). In between the sonication steps, the coverslips were rinsed with deionized water. Coverslips were dried overnight at 110 °C. PDMS and glass coverslips were plasma cleaned in an PE-50 plasma cleaner (PlasmaEtch, Carson City, NV, USA) using oxygen as gas for 1 min, prior to attachment of the PDMS to the glass. The plasma cleaning step results in a covalent bond between the glass and the PDMS.

### 2.6. Cell Culture

Peripheral blood monocytes were isolated from buffy coats, as described in Baranov et al. [23], from healthy donors. Buffy coats were obtained as fully anonymized specimens from the Dutch blood bank, Sanquin. The research of the Department of Molecular Immunology was approved by the ethics committee of Sanquin, and an access use agreement was signed (NVT0459.00; 15 June 2018). All blood donors were informed about the research and granted their consent to Sanquin. The research is exempt from ethical approval by the Dutch Medical Research with Human Subjects Law (non WMO research), because the investigators cannot discern the identity of the donors and the blood donors do not have to undergo extra procedures. Monocytes were then differentiated into monocyte-derived dendritic cells (moDCs) via incubation with interleukin (IL)-4 (300 U/mL) and granulocyte-macrophage colony-stimulating factor (GM-CSF; 450 U/mL) for 6 days, in RPMI (Roswell Park Memorial Institute medium) supplemented with 10% serum, antibiotics (100 μg/mL penicillin, 100 µg/mL streptomycin, and 0.25 µg/mL amphotericin B, Gibco), and 2 mM glutamine.

### 2.7. Confocal Microscopy

Cells were seeded onto glass coverslips or wells for at least 12 h, before fixing in 4% paraformaldehyde (PFA) for 15 min. Samples were next treated for 5 min in a 0.1% (*v*/*v*) Triton-X100 solution. Cells were blocked in a 20 mM glycine 3% BSA PBS-based solution for 1 h, prior to staining with phalloidin (Alexa Fluor 488) (A12379, Thermo Fisher, Waltham, Massachusetts, USA) and DAPI. Images were collected with a Zeiss LSM 800 (Jena, Germany) confocal microscope, equipped with a Plan-Apochromat (63×/1.4) oil DIC M27 (FWD = 0.19 mm) objective (Zeiss). Images were acquired using the Zeiss ZEN software (version 2.3, Stuttgart, Germany). DAPI was excited by a 405 nm laser, and Alexa Fluor 488 phalloidin was excited by a 488 nm laser. For Z-series, a slice interval of 0.31 µm was used.

### 2.8. ELISA

First, 150,000 moDCs were seeded in 500 µL of RPMI. The supernatant was collected following an overnight lipopolysaccharide (LPS) stimulation (1 μg/mL O111:B4 Sigma-Aldrich 32160405, St. Louis, MO, USA) treatment. Cytokine production by human moDCs cells was measured using a human TNF alpha uncoated ELISA kit (Invitrogen, 88-7346-88, Waltham, MA, USA) and a human IL-6 uncoated ELISA kit (Invitrogen, 88-7066-22, Waltham, MA, USA), according to the manufacturer’s guidelines.

## 3. Results

**Figure 1:**
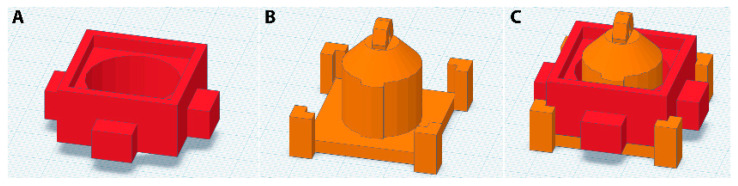
PLA mold design. (**A**) Design of the outer part. (**B**) Design of the inner part. (**C**) Superposition of the two parts as assembled.

**Figure 2:**
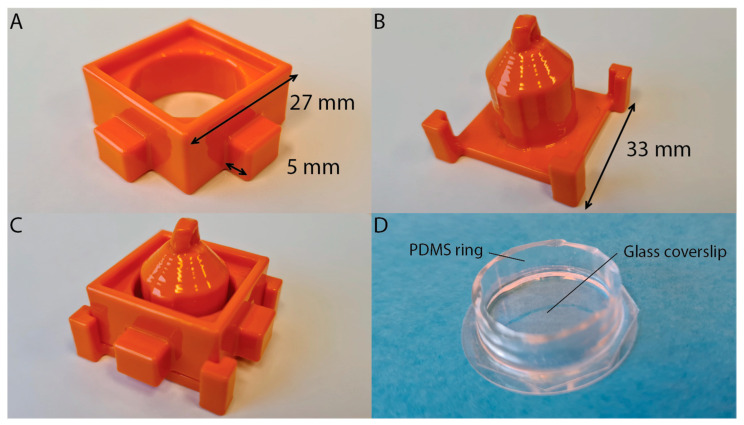
Final product mold and PDMS well. (**A**) Printed and smoothed outer part of the mold. Dimensions are added to the picture. The width and depth of the main structure is 27 × 27 mm^2^, with 5 mm protrusions on all sides. The height is 13 mm. (**B**) Printed and smoothed inner part of the mold. The width is added to the picture. The height of the structure is 27 mm and the cylindrical inner part has a diameter of 17.5 mm. (**C**) The printed mold parts assembled. (**D**) The well formed by attaching the PDMS ring onto a clean glass coverslip (diameter 27 mm). The PDMS ring measures 20 mm as outer ring diameter and 3 mm as height.

To study the ability of PLA to act as an effective PDMS mold, a mold was designed to create a well, designed for cell culturing and light microscopy studies. The mold was designed in two parts, for ease of releasing the mold from the PDMS. The outer part measures 37 × 37 × 13 mm^3^, including the protrusions (Figure 1A). This part consists of an inner ring of 20 mm in diameter, a raised rim to facilitate the casting of the PDMS prepolymer, and grips, to ease the removal of the mold (Figure 1A and Figure 2A). The inner part measures 33 × 33 × 27 mm^3^ (Figure 1B). This part consists of a 3 mm high surface and an inner cylinder of 17.5 mm in diameter, a segment of the inner cylinder has an extra 0.75 mm thick protrusion (Figure 1B and Figure 2B). The thicker protrusion was included to thin the wall of the PDMS ring at a specific location, to accommodate a thermocouple. By inserting a thermocouple into the experimental chamber, the buffer solution temperature in the well can be measured during the experiment. The inner mold also features four notched pillars at the corners, for alignment purposes, and a ring on top, for suspending the part during the smoothing procedure.

Upon printing, due to the layer-by-layer printing process, the products contain undesirable ridges on the exterior of the print. These ridges must first be removed, to produce smooth PDMS wells and to ensure an easy release from the mold. The PDMS structures that were produced from molds that were not smoothed exhibited rough surfaces, and these PDMS rings could not be sealed watertight to the glass coverslips. Furthermore, it was difficult to remove these rings from the molds. Therefore, smoothing of the molds was needed. In order to do this, we decided to use chemical post-processing to smooth the surface of the molds. Smoothing of 3D printed structures can be performed in various ways, including immersing the structures in acetone or chloroform solvents for a limited time [24,25]. Instead of using solvent immersion, we applied chloroform vapor to smooth our PLA-based 3D-printed molds. As PLA is partially soluble in chloroform, chloroform vapor is ideally suited for smoothing PLA [26]. Working under a fume hood, chloroform was heated in a large, covered beaker to produce the desired vapor. Polytetrafluoroethylene (PTFE) tubing was threaded through the ring of the inner part of the mold, to suspend that part in the vapor, while the outer part of the mold was suspended in the vapor using a clamp. The molds were suspended in the chloroform vapor four times, or until smooth, for a duration of 30 s. In between each cycle, a 1 min waiting time was taken into account. When smooth, the parts were left suspended in a fume hood at room temperature, for a minimum of 1 h, to dry. Afterward, the mold parts were placed in a vacuum desiccator overnight, to ensure the evaporation of any remaining chloroform. The mold was then assembled to form the PDMS well, placing the outer part over the inner (Figure 1C and Figure 2C). After assembly, the parts were secured in place using masking tape. The PDMS elastomer mixture was prepared (see Materials and Methods) and poured into the assembled mold. After desiccation, the PDMS was cured for at least 4 h, at 60 °C. Upon finishing of the curing process, the mold was disassembled and the PDMS ring was removed from the mold. Excess PDMS was removed using a scalpel, so the ring measured 3 mm in height. To construct the well, a clean glass coverslip and the PDMS ring were placed in an oxygen plasma cleaner for 1 min, and then pressed together by hand, for at least 1 min. This bound the PDMS to the glass in a water-tight manner. To test this, the well was tested for leakage by filling it with 800 µL of phosphate-buffered saline and leaving it at room temperature for 72 h. Typically, no sign of leakage was detected.

**Figure 3:**
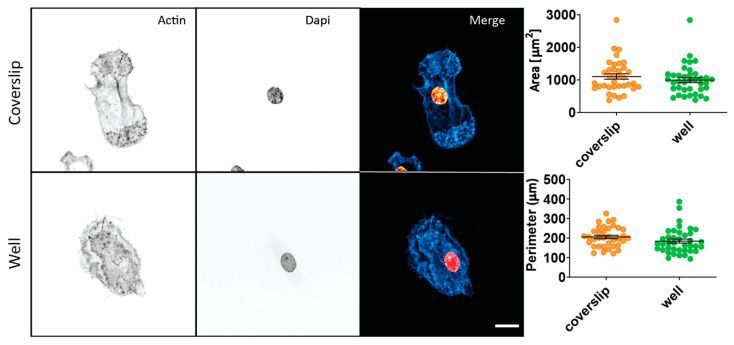
Fluorescence microscopy data of monocyte-derived dendritic cell (moDC) morphology. The morphologies of moDCs cultured on a glass coverslip or cultured in a PDMS well, were compared by confocal fluorescence microscopy imaging and analysis. Cells were stained with phalloidin (actin stain, cyan in merge) and DAPI (nucleus stain, orange). The area and perimeter of the cells were measured to give an indication of average morphology. These showed no significant difference between coverslip-cultured and well-cultured cells at the α = 0.05 level, as measured using an unpaired two-tailed *t*-test. The number of data points for the coverslip results is *n* = 36 and for the well it is *n* = 38, for both parameters. The results represent data from three healthy donors. Whiskers represent the standard error of the mean. Scale bar is 10 μm.

**Figure 4:**
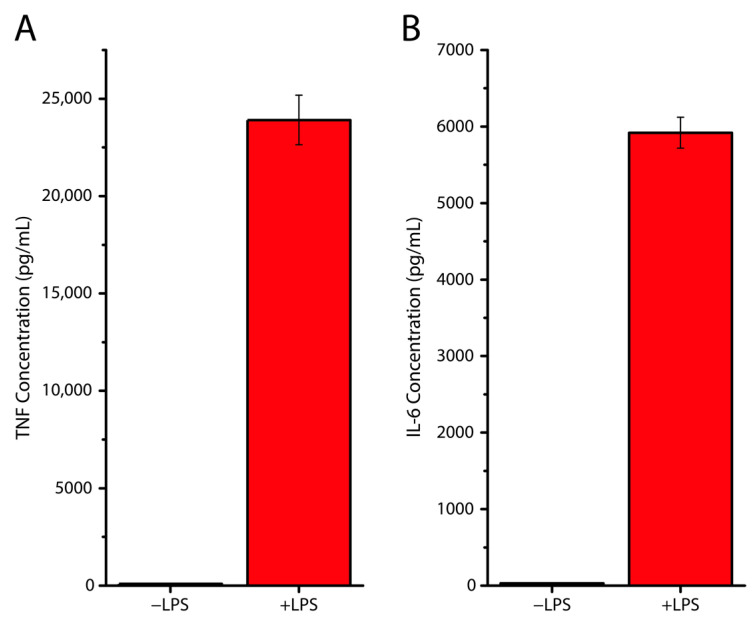
LPS-stimulation dependent cytokine production in PDMS wells. (**A**) Column graphs showing TNF cytokine production in non-LPS-stimulated and LPS-stimulated cells, cultured in PDMS wells. The data shows ELISA results when testing for TNF production. (**B**) Column graphs showing IL-6 cytokine production in non-LPS-stimulated and LPS-stimulated cells, cultured in PDMS wells. The data shows ELISA results when testing for IL-6 production. Whiskers represent the standard error of the mean. For both TNF and IL-6 measurements, a significant difference was found between −LPS and +LPS conditions at the α = 0.05 level, as measured using an unpaired two-way *t*-test.

To examine the viability of its use in live cell experiments, eukaryotic cells were used. In particular, these were monocyte-derived dendritic cells (moDCs). Monocytes are a type of white blood cells and are part of the mammalian immune system. These cells can differentiate into dendritic cells, which are then referred to as moDCs. The moDCs that were used in this study, were cultured under three different conditions. A glass coverslip was placed in (i) a well of a standard 6-well plate, (ii) the PDMS well, and (iii) the PDMS well with added lipopolysaccharide (LPS). LPS is a major surface molecule of Gram-negative bacteria. The LPS condition was added as a positive control for immune response, as LPS is known to trigger a strong immune response in moDCs. This leads to moDC maturation and the release of cytokines (proteins that have an effect on the immune system), amongst which are TNF and IL-6 [27,28,29]. After overnight culturing, the cells were examined for significant morphological changes by fluorescence microscopy, and tested for cytokine production using ELISA. Morphological studies found no significant difference between the area and perimeter of the PDMS well-cultured cells when compared to ‘standard’ coverslip-cultured cells (Figure 3). In addition, the ELISA experiments showed that non-LPS-stimulated moDCs showed negligible cytokine production, when compared to LPS-stimulated moDCs (Figure 4). This showed that the cells stayed alive in the wells, without showing any additional reaction to the PDMS well.

## 4. Discussion

A PDMS well structure was chosen for the proof-of-concept design, as a relatively cheap and easy-to-produce alternative, for a commercial biological microscopy sample holder. The final product was well suited for this purpose and the mold was re-usable. The PDMS-glass well proved a functional, easily produced, reusable well, for use in, amongst other things, live cell microscopy. Morphological studies and cytokine assays, showed that the PDMS well did not significantly affect the culturing of the moDCs. This suggests that any trace amounts of PLA left in the PDMS are unlikely to affect live cell studies performed using PLA mold-formed PDMS constructs. This confirmed the expectations as, not only is PLA considered GRAS by the FDA, it is also a polymer known to be relatively biocompatible and inert [21]. As such, PLA has proven to be a viable candidate as a general-use 3D printing material, for instance, for PDMS molds. PLA is cheap, it can be printed quickly and it is straightforward to handle. This makes PLA molds well suited for the use of rapid prototyping PDMS structures, for use with live-cell studies or other (micro)biological and biochemical applications. This being said, PLA printing does have its limitations. Not only does filament printing inherently have a lower resolution than photolithography, but the vast majority of general-use filament printers also have a lower resolution than the average resin printer. The z resolution of filament printers is generally around 0.1 mm, and the x/y resolution leads to a minimum structure size of ~0.5 mm. When a smoothing step is applied to optimize the wall structure of the mold, this resolution is even lower. Nevertheless, filament printing is a much more accessible technology than photolithography or resin printing. Furthermore, many structures, including wells for live cell culturing and fluorescence observations, do not require very fine details. So, while other polymer 3D printing techniques, such as vat photopolymerisation or material jetting processes can be used [30,31], PLA printing has specific advantages. Next to PLA being cheaper than resins, there is another advantage. Unlike common resin types used in vat polymerization printers, PLA does not inhibit the curing of the PDMS prepolymer [32].

## 5. Conclusions

This study shows that, the widely used method of filament 3D printing of PLA, despite its relative underrepresentation in the scientific literature, is highly viable for use in the rapid prototyping of molds to fabricate PDMS structures. PLA filament printing is particularly underrepresented in the field of live-cell studies, though this study shows that it is a perfectly viable approach and that matters concerning printing resolution and surface roughness do not need to be an issue. Therefore, filament 3D printing with PLA is expected to find its place in research and development for the easy, cheap, and fast production of molds and parts that do not inhibit the curing of PDMS.

## Data Availability

Data used in this study will be provided upon reasonable request.

## Supplementary Materials

The following supporting information can be downloaded at: https://www.mdpi.com/article/10.3390/polym15061498/s1.

## References

[1] Mata A., Fleischman A.J., Roy S. (2005). Characterization of polydimethylsiloxane (PDMS) properties for biomedical micro/nanosystems. Biomed. Microdevices.

[2] McDonald J.C., Whitesides G.M. (2002). Poly(dimethylsiloxane) as a material for fabricating microfluidic devices. Acc. Chem. Res..

[3] Xia Y., Whitesides G.M. (1998). Soft Lithography. Angew. Chem. Int. Ed. Engl..

[4] Folch A., Toner M. (1998). Cellular micropatterns on biocompatible materials. Biotechnol. Prog..

[5] Roos W., Ulmer J., Gräter S., Surrey T., Spatz J.P. (2005). Microtubule gliding and cross-linked microtubule networks on micropillar interfaces. Nano Lett..

[6] Fitzgerald M.L., Tsai S., Bellan L.M., Sappington R., Xu Y., Li D. (2019). The relationship between the Young’s modulus and dry etching rate of polydimethylsiloxane (PDMS). Biomed. Microdevices.

[7] da Silva E.N.T., Ferreira V.S., Lucca B.G. (2019). Rapid and inexpensive method for the simple fabrication of PDMS-based electrochemical sensors for detection in microfluidic devices. Electrophoresis.

[8] Hattori K., Sugiura S., Kanamori T. (2014). Microfluidic perfusion culture. Methods Mol. Biol..

[9] Siddique A., Meckel T., Stark R.W., Narayan S. (2017). Improved cell adhesion under shear stress in PDMS microfluidic devices. Colloids Surf. B Biointerfaces.

[10] Roos W.H., Campas O., Montel F., Woehlke G., Spatz J.P., Bassereau P., Cappello G. (2008). Dynamic kinesin-1 clustering on microtubules due to mutually attractive interactions. Phys. Biol..

[11] Kim J., An H., Seo Y., Jung Y., Lee J.S., Choi N., Bong K.W. (2017). Flow lithography in ultraviolet-curable polydimethylsiloxane microfluidic chips. Biomicrofluidics.

[12] Amin R., Knowlton S., Hart A., Yenilmez B., Ghaderinezhad F., Katebifar S., Messina M., Khademhosseini A., Tasoglu S. (2016). 3D-printed microfluidic devices. Biofabrication.

[13] Bonyár A., Sántha H., Varga M., Ring B., Vitéz A., Harsányi G. (2012). Characterization of rapid PDMS casting technique utilizing molding forms fabricated by 3D rapid prototyping technology (RPT). Int. J. Mater. Form..

[14] Hwang Y., OPaydar H., Candler R.N. (2015). 3D printed molds for non-planar PDMS microfluidic channels. Sens. Actuators A Phys..

[15] Mi S., Du Z., Xu Y., Sun W. (2018). The crossing and integration between microfluidic technology and 3D printing for organ-on-chips. J. Mater. Chem. B.

[16] Pang X., Zhuang X., Tang Z., Chen X. (2010). Polylactic acid (PLA): Research, development and industrialization. Biotechnol. J..

[17] Singhvi M.S., Zinjarde S.S., Gokhale D.V. (2019). Polylactic acid: Synthesis and biomedical applications. J. Appl. Microbiol..

[18] Li G., Zhao M., Xu F., Yang B., Li X., Meng X., Teng L., Sun F., Li Y. (2020). Synthesis and Biological Application of Polylactic Acid. Molecules.

[19] Okolie O., Stachurek I., Kandasubramanian B., Njuguna J. (2020). 3D Printing for Hip Implant Applications: A Review. Polymers.

[20] Prasad L.K., Smyth H. (2016). 3D Printing technologies for drug delivery: A review. Drug Dev. Ind. Pharm..

[21] DeStefano V., Khan S., Tabada A. (2020). Applications of PLA in modern medicine. Eng. Regen..

[22] Alexander A.E., Wake N., Chepelev L., Brantner P., Ryan J., Wang K.C. (2021). A guideline for 3D printing terminology in biomedical research utilizing ISO/ASTM standards. 3D Print Med..

[23] Baranov M.V., Ter Beest M., Reinieren-Beeren I., Cambi A., Figdor C.G., van den Bogaart G. (2014). Podosomes of dendritic cells facilitate antigen sampling. J. Cell Sci..

[24] Hambali R.H., Cheong K.M., Azizan N. (2017). Analysis of the influence of chemical treatment to the strength and surface roughness of FDM. IOP Conference Series: Materials Science and Engineering.

[25] Valerga A.P., Batista M., Fernandez-Vidal S.R., Gamez A.J. (2019). Impact of Chemical Post-Processing in Fused Deposition Modelling (FDM) on Polylactic Acid (PLA) Surface Quality and Structure. Polymers.

[26] Salazar R., Pizarro F., Vasquez D., Rajo-Iglesias E. (2022). Assessment of 3D-printed waveguides using conductive filaments and a chloroform-based smoothing process. Addit. Manuf..

[27] Barrientos L., Bignon A., Gueguen C., de Chaisemartin L., Gorges R., Sandré C., Mascarell L., Balabanian K., Kerdine-Römer S., Pallardy M. (2014). Neutrophil extracellular traps downregulate lipopolysaccharide-induced activation of monocyte-derived dendritic cells. J. Immunol..

[28] Rajnavolgyi E., Laczik R., Kun V., Szente L., Fenyvesi É. (2014). Effects of RAMEA-complexed polyunsaturated fatty acids on the response of human dendritic cells to inflammatory signals. Beilstein. J. Org. Chem..

[29] Rana D., Duseja A., Dhiman R.K., Chawla Y., Arora S.K. (2013). Maturation defective myeloid dendritic cells in nonalcoholic fatty liver disease patients release inflammatory cytokines in response to endotoxin. Hepatol. Int..

[30] Khan Z., Albalawi H., Valle-Perez A., Aldoukhi A., Hammad N., Herrera Ponce de Leon E., Abdelrahman S., Hauser C. (2022). From 3D printed molds to bioprinted scaffolds: A hybrid material extrusion and vat polymerization bioprinting approach for soft matter constructs. Mater. Sci. Add. Manuf..

[31] Goh G.D., Sing S.L., Lim Y.F., Thong J.L.J., Peh Z.K., Mogali S.R., Yeong W.Y. (2021). Machine learning for 3D printed multi-materials tissue-mimicking anatomical models. Mater. Design.

[32] Venzac B., Deng S., Mahmoud Z., Lenferink A., Costa A., Bray F., Otto C., Rolando C., Le Gac S. (2021). PDMS Curing Inhibition on 3D-Printed Molds: Why? Also, How to Avoid It?. Anal. Chem..

